# Exploring the Sustainable Benefits of Adherence to the Mediterranean Diet during the COVID-19 Pandemic in Italy

**DOI:** 10.3390/nu15010110

**Published:** 2022-12-26

**Authors:** Paola Gualtieri, Marco Marchetti, Giulia Frank, Rossella Cianci, Giulia Bigioni, Carmela Colica, Laura Soldati, Alessandra Moia, Antonino De Lorenzo, Laura Di Renzo

**Affiliations:** 1 Section of Clinical Nutrition and Nutrigenomics, Department of Biomedicine and Prevention, University of Tor Vergata, Via Montpellier 1, 00133 Rome, Italy; 2 School of Specialization in Food Sciences, University of Rome Tor Vergata, Via Montpellier 1, 00133 Rome, Italy; 3 Department of Translational Medicine and Surgery, Catholic University of the Sacred Heart, Fondazione Policlinico Universitario A. Gemelli, IRCCS, 00168 Rome, Italy; 4 Department of Physic, University of Rome Sapienza, Piazza Aldo Moro 5, 00185 Rome, Italy; 5 CNR, IBFM UOS, Università Magna Graecia, Viale Europa, 88100 Germaneto, Italy; 6 Department of Health Sciences, University of Milan, 20122 Milan, Italy; 7 Department of Biomedicine and Prevention, University of Rome Tor Vergata, 00133 Rome, Italy

**Keywords:** Mediterranean diet, food sustainability, food cost, organic and conventional food, lifestyle

## Abstract

This study aimed to identify adherence to the Mediterranean diet (MedDiet) and its effect on health and environmental and socioeconomic sustainability during the COVID-19 pandemic among a sample of the Italian population. Notably, it intended to assess the effect of adherence to the MedDiet on ecological footprints and food expenditure. A survey was conducted from the 5th to the 24th of April 2020 on Google Forms. The MEDAS questionnaire was used to determine the level of adherence to the MedDiet. The carbon footprint (CO_2_), water footprint (H_2_O), and food cost were calculated. In total, 3353 participants completed the questionnaire, ranging from 18 to 86 years old. A statistically significant difference was observed in the CO_2_ and H_2_O among BMI groups (*p* < 0.001). The low- and medium-MEDAS groups showed higher CO_2_ (*p* < 0.001). The food cost (EUR/week) resulted in statistically significant differences among the MEDAS groups. The CO_2_ results were significantly lower in organic-market buyers compared to non-organic-market buyers (*p* < 0.001). Public health must promote awareness of how adhering to a healthy lifestyle and making appropriate food choices can positively impact our health and social and economic well-being.

## 1. Introduction

Sustainable diet (SD) and sustainable nutrition are concepts that were described for the first time in 1986 by Gussow and Clancy [1] as a diet made up of healthy foods that contribute to the sustainability of the entire nutrition system.

Today, SDs are defined as diets with “low environmental impacts that contribute to food and nutrition security and healthy life for present and future generations. SD are protective and respectful of biodiversity and ecosystems, culturally acceptable, accessible, economically fair and affordable; nutritionally adequate, safe and healthy; while optimizing natural and human resources” [2].

Diets connect human health and environmental well-being. A healthy and environmental SD should include nutritional adequacy, availability and affordability, sociocultural well-being, resilience, food safety, and waste and loss reduction [3].

The dietary composition can contribute positively to environmental and human health outcomes. Plant-based diets limit meat consumption, though the levels and types of animal sources can vary depending on the diet (e.g., vegetarian and Mediterranean) [3]. Diets that are high in calories, added sugars, saturated fats, processed foods, and red meats are less environmentally sustainable than healthy plant-based diets associated with reduced greenhouse gas (GHG) emissions and land and water use.

The selection of raw materials is fundamental. Indeed, healthy foods include whole grains, fruits, vegetables, legumes, nuts [4], seeds, fish, and seafood. Unhealthy foods include excessive amounts of unprocessed red meats, processed meats (cured and salted), overly processed starches, simple sugars, sugar-sweetened beverages, and foods containing high amounts of saturated fat, trans fat, dietary cholesterol, and sodium [5,6].

The Mediterranean diet (MedDiet) is not only a sustainable food model that includes elements recognized as distinctive, such as biodiversity; seasonality; culinary activities; and traditional, local, and ecofriendly food products, but also a lifestyle characterized by conviviality, adequate rest, and regular physical activity [7,8].

A high intake of vegetables, fruits, nuts, cereals, whole grains, and olive oil; moderate consumption of fish and poultry; and low quantities of sweets, red meat, and dairy products characterize the MedDiet. There is a low saturated fat intake and a higher monounsaturated fat intake. Therefore, the MedDiet provides high amounts of fiber, glutathione, and antioxidants and has a balanced ratio of omega-6/omega-3 essential fatty acids [9].

MedDiet adherence is correlated with a significant improvement in health status [10] and increasing well-being [11]. The MedDiet is linked to a positive mental status and healthier body composition, promoting reductions in weight, waist circumference [12], fat mass, and body mass index (BMI) [13] that are even related to the mean blastocyst euploidy rate [14]. Furthermore, it has good effects on metabolic abnormalities, leading to lower incidences of metabolic syndrome and type 2 diabetes [15]. These improvements reflect beneficial changes in central obesity, atherogenic dyslipidemia, hypertension, and insulin resistance. Adherence to the MedDiet is associated with better maternal and newborn outcomes [16]. Furthermore, the MedDiet may reduce the morbidity of major chronic diseases, positively influencing the aging process, decreasing inflammation, and improving endothelial function and respiratory fitness [17].

At the beginning of March 2020, due to the COVID-19 pandemic, the Italian Government decided to adopt for more stringent containment measures: a ban on mass gatherings and events and on meeting up without an urgent reason was issued for the entire national territory. A strong impact on the eating habits and lifestyle of Italians was observed [18] due to the COVID-19 lockdown, but the impacts on health and environmental and socioeconomic sustainability were not investigated.

The purpose of this study was to calculate the environmental, socioeconomic, and health impacts of the Italian population’s eating habits and lifestyle during the COVID-19 pandemic, verifying the effect of adherence to the MedDiet on ecological footprints and food expenditure.

## 2. Materials and Methods

### 2.1. Survey Methodology

A survey was conducted among the Italian population from the 5th to the 24th of April 2020 using the “Eating Habits and Lifestyle Changes in COVID-19 lockdown” (EHLC-COVID19) questionnaire [18] on an online platform, Google Form, that was accessible through any device with an internet connection. This method of administration provided a statistical collective whose population parameters could not be controlled, as is the case for probabilistic sampling.

The EHLC-COVID19 questionnaire consisted of 43 questions divided into four different sections: biographical data (age, gender, hometown, and current occupation), anthropometric information (self-reported weight and height), information on dietary habits through the Mediterranean diet adherence screener (MEDAS) [19] and a questionnaire on daily food consumption and food frequency (FFQ) [20]; and information on lifestyle habits (expenditure, smoking habits, hours of sleep, physical activity, and organic or conventional market purchases).

BMI was calculated with the formula BMI = weight (kg)/Height (m^2^).

The population was divided according to BMI: underweight, BMI ≤ 18.49 kg/m^2^; normal weight, BMI 18.5–24.99 kg/m^2^; pre-obese, BMI 25–29.99 kg/m^2^; and obese, BMI ≥ 30 Kg/m^2^ [21].

From the MEDAS score, the participants were divided into three bands: low adherence (0–5 points), medium adherence (6–9 points), and high adherence (≥10 points) to the Mediterranean diet [19].

This research was carried out strictly in accordance with national and international standards and the Declaration of Helsinki (2000). Before participating in the study, all participants were fully informed about the study’s objectives and were required to approve the consent form. Participants filled out a survey that was directly linked to the Google platform. Participants’ personal information was anonymized, preventing sensitive personal data from being traced and protecting and preserving confidentiality.

### 2.2. Environmental and Economic Impact Analyses

All data collected through the survey were exported into an Excel spreadsheet and analyzed from an environmental and economic perspective. In particular, food types and quantities were derived from the MEDAS and FFQ data. We derived daily or weekly quantities from specific questions in the questionnaire, as the participants explicitly reported these. The study of quantities was then carried out from the perspective of weekly consumption. It was then possible to assess the carbon footprint [22] and the water footprint [23], differentiating them into organic and non-organic consumption. On the platform provided by the WWF [24], the CO_2_ emissions (CO_2_) and H_2_O consumption (H_2_O) due to the production of organic and non-organic food per package (with expressed weight) were reported for the different foods. The CO_2_ and H_2_O values were derived for each participant using the proportions. On the ISTAT price database of April 2020 [25], the average costs in Italy in April 2020 (with expressed reference weights) were given for the different foods. The cost of each participant’s expenditure was derived using the proportions.

### 2.3. Statistical Analyses

The collected data were analyzed for the presence of outliers, and the Shapiro–Wilk test was performed to evaluate the variable distribution.

Bartlett’s or Levene’s tests were used to test variances’ homogeneity. The presented data are expressed as means, medians, standard deviations, and minimum and maximum values.

Pearson correlation coefficients were calculated to evaluate the correlations between continuous variables. At the same time, the chi-square test was employed to assess the associations between categorical variables.

The Mann–Whitney U and Kruskal–Wallis tests were performed to compare continuous variables among two or more groups.

Linear regression analyses with the stepwise method were conducted to investigate the associations between variables. In contrast, a binary multinomial logistic regression was performed to investigate the associations between categorical variables (dependent) and continuous or categorical variables (independent). Finally, a generalized linear model (GLM) was created to investigate the association and future prediction of categorical variables (dependent) with continuous or categorical variables (independent). The results were significant for *p*-values < 0.05. The statistical analysis was performed using R (CRAN, Rcmdr package, vers. 2.7-1).

## 3. Results

### 3.1. The Interplay between BMI, Employment, and Environmental Impact

The general characteristics and anthropometrics of the population are reported in Table 1. A total of 3353 participants completed the questionnaire, ranging from 18 to 86 years.

To evaluate the correlation between CO_2_ (expressed as eq/week) and BMI, the whole sample was stratified into four groups: (1) underweight, (2) normal weight, (3) pre-obese, and (4) obese. According to BMI, 4% of the subjects were underweight; 63.5% of the subjects were normal weight; 23% of the subjects were pre-obese; and 9.5% of the subjects were obese.

The Kruskal–Wallis rank sum test showed a statistically significant difference in the CO_2_ among the four BMI groups (*p* < 0.001). In particular, with the post hoc analysis, the normal weight group’s food consumption resulted in a lower CO_2_ than the pre-obese group (*p* = 0.01). The underweight group’s food consumption showed a lower CO_2_ compared to the normal weight, pre-obese, and obese groups (*p* = 0.004, *p* < 0.001, and *p* < 0.001, respectively) (Table 2). Between the normal weight and obese group, no differences were found (*p* = 0.3).

Moreover, the H_2_O (expressed as L/week) showed statistically significant differences among the four BMI groups through the Kruskal–Wallis rank sum test (*p* < 0.001). Notably, the normal weight group showed a lower H_2_O than the pre-obese and obese groups (*p* = 0.01 and *p* = 0.006, respectively). Furthermore, the underweight group exhibited a lower H_2_O compared to the normal weight, pre-obese, and obese groups (*p* = 0.009, *p* < 0.001, and *p* < 0.001, respectively) (Table 2).

Using a generalized linear model (GLM), a higher BMI was correlated with elevated CO_2_ and H_2_O in terms of future prediction (Table 3).

The Kruskal–Wallis rank sum test for CO_2_ showed statistically significant differences among the occupation groups (*p* < 0.001). With the post hoc analysis, students showed a lower CO_2_ than the unemployed, employed, and retired groups (*p* = 0.001, *p* < 0.001, and *p* < 0.001, respectively). Additionally, the employed group resulted in a lower CO_2_ than the retired group (*p* = 0.006), and the unemployed group resulted in lower CO_2_ compared to the retired group (*p* = 0.02). No other differences were found among the four groups (Table 4).

Subsequently, the H_2_O also resulted in statistically significant differences among the employment groups through the Kruskal–Wallis rank sum test (*p* < 0.001). Across the post hoc analyses, students showed a lower H_2_O compared to the unemployed, employed, and retired groups (*p* < 0.001, *p* < 0.001, and *p* < 0.001, respectively). Furthermore, the employed resulted in a lower H_2_O compared to the retired group (*p* = 0.001), and the unemployed group resulted in a lower H_2_O than the retired group (*p* = 0.01). No other differences were found among the four groups (Table 4).

### 3.2. The Interplay between Employment, Environmental Impact, and MEDAS

The whole sample was stratified into three classes of adherence to the MedDiet based on the MEDAS values. In total, 21.6% showed a low commitment, 63.1% showed a medium adherence, and 15.3% indicated a high adherence.

Regarding CO_2_ and its relationship with adherence to the MedDiet, the Kruskal–Wallis rank sum test showed a statistically significant difference in the CO_2_ among the three groups of adherence to the MedDiet (*p* < 0.001). In particular, with the post hoc analysis, the low- and medium-adherence groups showed higher CO_2_ compared to the high-adherence group (*p* < 0.001 and *p* < 0.001, respectively), and the medium-adherence group resulted in a more elevated CO_2_ in comparison to the low-adherence group (*p* < 0.001) (Table 5).

As for the CO_2_, the H_2_O also resulted in statistically significant differences among the three groups of adherence to the MedDiet, according to the Kruskal–Wallis rank sum test (*p* < 0.001). Specifically, with the post hoc analysis, the low-adherence and medium-adherence groups showed higher H_2_O compared to the high-adherence group (*p* < 0.001 and *p* < 0.001, respectively), and the medium-adherence group resulted in a higher H_2_O compared to the low-adherence group (*p* < 0.001) (Table 5).

Concerning the possible connection between the MedDiet and employment status, the Kruskal–Wallis rank sum test showed a statistically significant difference among the four employment status groups (*p* = 0.02). However, with the post hoc analysis, the only significant difference was for the students, with a higher MEDAS score than the unemployed group (*p* = 0.02); no other differences were found among the remaining groups (Table 6).

### 3.3. The Interplay between BMI, MEDAS, and Food Cost

A multiple linear regression based on the stepwise method showed that a higher MEDAS score was associated with decreases in CO_2_ and H_2_O (*r*^2^ = 0.97, *p* = 0.01; *r*^2^ = 0.87, *p* < 0.001, respectively) (Table 7).

The weekly food cost (expressed as EUR/week) resulted in statistically significant differences among the MEDAS groups. By performing the Kruskal–Wallis rank sum test, we found a significant difference in the weekly food cost among the MEDAS groups (*p* < 0.001). With the post hoc analysis, we found the high-adherence group had lower weekly food costs in comparison with the low- and medium-adherence groups (*p* < 0.001 and *p* < 0.001, respectively), and the medium-adherence group had lower weekly food costs compared to the low-adherence group (*p* = 0.004) (Table 8).

### 3.4. The Interplay between the Organic Market, MEDAS, and Environmental Impact

The Mann–Whitney U test showed that the MEDAS score was statistically higher in organic-market buyers compared to non-organic-market buyers (*p* < 0.001) (Table 9).

The CO_2_ results were significantly lower in the organic-market buyers compared to the non-organic-market buyers (*p* < 0.001), and on the other hand, the weekly food costs were statistically higher for the organic-market buyer group (*p* < 0.001). No significant changes were observed for the H_2_O between these groups (Table 9).

A binary logistic regression confirmed an enhanced MEDAS score was associated with the organic-market buyers (OR = 2.1, *p* < 0.001) and highlighted a lower CO_2_ for the organic-market buyers (OR = 0.12, *p* < 0.001).

## 4. Discussion

This study provides valuable insights into adherence to the MedDiet and its impact on footprints and food costs during the COVID-19 pandemic and subsequent lockdowns. Our study showed statistically significant differences in the CO_2_ and H_2_O among the four BMI groups (*p* < 0.001 and *p* < 0.001, respectively). We demonstrated that the low- and medium-adherence groups showed a higher CO_2_ and a higher H_2_O than the high-adherence group (*p* < 0.001 and *p* < 0.001, respectively). In the same way, the medium-adherence group resulted in a higher CO_2_ and a higher H_2_O than the low-adherence group (*p* < 0.001). Furthermore, a lower BMI was associated with decreased CO_2_ and H_2_O. Food costs resulted in statistically significant differences among the MEDAS groups. Indeed, the high-adherence group had a lower weekly food cost in comparison with the low- and medium-adherence groups (*p* < 0.001 and *p* < 0.001, respectively), and the medium-adherence group presented a lower weekly food cost compared to the low-adherence group (*p* = 0.004). The MEDAS score was statistically higher in organic-market buyers compared to non-organic-market buyers (*p* < 0.001). The CO_2_ results were significantly lower for organic-market buyers compared to non-organic-market buyers (*p* < 0.001).

Several studies have shown that a healthy eating model is based on a diet rich in vegetables, legumes, cereals, and fruit [26,27,28]. Differences in the consumption of healthy foods regarding demographic data such as age, ethnicity [29,30], education [31], and socioeconomic factors [32,33] were highlighted.

As populations have changed their nutritional habits, choosing nutrient-poor and energy-dense foods, there have been global increases in BMI and the incidence of obesity since the first years of life. This finding is alarming, as obesity is a multifactorial and complex disease that significantly impacts physical and psychosocial health, affecting the quality of life [34,35].

Denoth et al., in a sample of 33,127 subjects participating in the Italian population IPSAD(^®^)2011 survey, highlighted low consumption of MedDiet patterns among youth and the frequent association of sociocultural and psychological issues with an inappropriate lifestyle with obesity [36].

According to our data, and disagreeing with Denoth et al. [36], during the COVID-19 pandemic, students (19.1%) showed lower values of BMI and footprints compared to the unemployed (8.9%), employed (68.2%), and retired (4.5%) groups (*p* < 0.001, *p* < 0.001, and *p* < 0.001, respectively). Moreover, the employed resulted in lower food consumption at high levels of CO_2_ and H_2_O compared to the unemployed and retired groups. In particular, the consumption data show that retired individuals have higher footprints that are linked to higher food consumption since the MEDAS results are not significantly different.

It has been shown that the food environment around us can lead to a nutritional transition, and proximity to a supermarket or fast food is associated with increased BMI [37]. Furthermore, although several studies have shown reductions in CO_2_ with the MedDiet, increases in costs were observed in comparison with other diets [38,39,40].

Therefore, the MedDiet has been proposed to be at the center of health and sustainability policies. We demonstrated that groups with low and medium adherence to the MedDiet showed a higher CO_2_ and a higher H_2_O than the high-adherence group. In the same way, the medium-adherence group resulted in a higher CO_2_ and a higher H_2_O than the low-adherence group. Furthermore, a lower BMI was associated with decreased CO_2_ and H_2_O.

For the first time, we found that during the COVID-19 lockdown the high-adherence group had a lower weekly food cost in comparison with the low- and medium-adherence groups, and the medium-adherence group presented a lower weekly food cost compared to the low-adherence group. Therefore, our results show that greater adherence to the MedDiet is affordable and convenient. This is crucial, as affordability is the first step in fighting food insecurity [40], which affects about 800 million people [41].

Food consumption is based on the relationships between different players in the chain: producers, distributors, and consumers, so the contemporary diet is no longer sustainable, as it is composed of foods whose production is energy-intensive and has an impact on the environment, requiring vast tracts of land, which could exacerbate other problems related to food production and supply [42,43]. However, it is known that food production is the most significant cause of environmental and climate change globally [44]. The food chain contributes to GHG emissions [45], occupies 40% of available land, uses 70% of available freshwater, and represents the largest driver of biodiversity loss, species extinction, and natural resource degradation [46].

SD, which is ecosystem-specific, offers a practical way of applying sustainability to food security and nutrition [47,48]. The food system involves steps such as agriculture, animal husbandry, production, processing, distribution, supply, marketing, preparation, and the consumption of food and beverages [48]. In recent years, organic farming and organic agriculture have been introduced. They refer to consumption that is more attentive to the environment and food health and are intended to increase the integrity and quality of nutritional characteristics [49,50].

In this context, our previous results affirmed that consuming organic foods within the Mediterranean dietary model maintains good health [51]. It is known how the Italian Mediterranean organic diet impacts health positively compared to a conventional diet in terms of reducing the inflammatory state and endothelial dysfunction associated with obesity, kidney diseases, the incidence of cardiovascular diseases, and the general development of chronic degenerative diseases [52]. According to European Union legislation (EU; No. 834/2007, No. 889/2008, No. 1235/2008, No. 848/2018, and No. 2047/2022) on organic farming and other regulations that apply in Italy, shops or supermarkets defined as organic can only sell organic food products. However, during the period of the COVID-19 pandemic, in terms of weekly shopping, only 13.9% of the participants bought from an organic market and therefore consumed organic food.

Our results showed that the MEDAS score was statistically higher in the organic-market buyers compared to non-organic-market buyers. Moreover, the CO_2_ results were significantly lower in the organic-market buyers compared to the non-organic-market buyers.

Based on our data, it is necessary to raise the population’s awareness [53] that greater consumption of organic foods has a lower carbon footprint. Furthermore, it has been shown [54] that organic foods contain more polyphenols and omega-3, positively influence the plasma levels of micronutrients and fatty acids, and positively affect the microbiota, suggesting that organic foods could increase the immune response [55,56] and could play an important role in obese patients affected by COVID-19 [57].

This study presented some limitations. The recruitment period was extended for a limited period to obtain adequate and prompt adherence to the survey in the first period of the COVID-19 pandemic. Data were self-reported or self-measured due to the lockdown.

Another limitation was represented by the fact that the respondents to the survey were mainly women (76%). This was probably due to the fact that women, as reported by the Italian National Institute of Statistics [58], participate more in social media and inform themselves more on the web and social networks about aspects concerning their health compared to men [59].

Age and employment as well as socioeconomic status, depending on the COVID-19 pandemic, must be considered as factors impacting MedDiet adherence. Furthermore, CO_2_, H_2_O, and food costs were estimated data, as it was impossible to carry out real measurements or have individual shopping receipts.

Despite the above limitations, this study had some strengths. This research was the first to investigate the effect of adherence to the MedDiet on footprints and food costs using individual-level data from an Italian survey [18]. Most importantly, the study used data from a large and recent sample of adults during the COVID-19 pandemic.

The COVID-19 lockdown and closures imposed by governments around the world have impacted nutritional behaviors in a significant number of people. Our findings on the environmental, economic, and health sustainability of the MedDiet during the COVID-19 pandemic should be used to guide public health policymakers to provide any nutritional advice in a new vision from a One Health perspective.

## 5. Conclusions

In conclusion, for the first time, the current study investigated the socioeconomic and environmental aspects of Italian eating habits during the COVID-19 pandemic period. Moreover, the health impact of food choices was analyzed. Differences in BMI, weight, MEDAS score, weekly food cost, CO_2_, and H_2_O among participants who bought from organic and non-organic markets were highlighted, and more extraordinary efforts are warranted to prevent an increase in the BMI of the population.

What emerged from this study is that higher adherence to the MedDiet plays a crucial role in citizen health without overlooking its economic and environmental sustainability.

Therefore, more excellent promotion of a healthy diet, characterized not only by the selection of healthy foods but also by a conscious and sustainable choice of foods, looking at the footprints, and more targeted policies must be pursued.

Adopting the MedDiet, on one hand, would be beneficial from a public health perspective; on the other hand, it would be a concrete measure of intervention in terms of environmental and economic sustainability [9].

## Figures and Tables

**Table 1:** General characteristics of the whole sample. Values are expressed as means and standard deviations (M ± SD) for continuous variables. Abbreviations: body mass index (BMI).

Parameters	Whole Sample (*n* = 3353)
Gender (F)	2689 (76.1)
Gender (M)	664 (23.9)
Age	36.0 [18.0–86.0] 38.5 ± 14.2
18–30 years	1228 (34.8)
31–50 years	1492 (42.2)
51–65 years	693 (19.6)
>66 years	120 (3.4)
Weight (kg)	65.0 [57.0–75.0] 67.34 ± 14.2
Height (cm)	166.0 ± 12.0
BMI (kg/m^2^)	24.00 ± 4.27
Underweight (≤18.4 kg/m^2^)	142 (4.0)
Normal weight (18.5–24.9 kg/m^2^)	2243 (63.5)
Pre-obese (25.0–29.9 kg/m^2^)	814 (23.0)
Obese	334 (9.5)
Unemployed (38.75 y)	289 (8.2)
Retired (67.25 y)	159 (4.5)
Students (21.54 y)	674 (19.1)
Employed (41.30 y)	2411 (68.2)
Purchase in the organic market	137 (3.9)
Purchase in the non-organic market	3396 (96.1)

**Table 2:** Comparisons between the BMI groups for CO_2_ and H_2_O. Values are expressed as median, minimum, and maximum values (median ± [min.–max. value]) for continuous variables.

	CO_2_ (eq/Week)		H_2_O (L/Week)	
BMI Groups				
	Median ± [minimum–maximum value]	*p*-value	Median ± [minimum–maximum value]	*p*-value
Overall population		<0.001 ***		<0.001 ***
Normal weight vs. underweight	22.1 ± [8.9–35.1] vs. 20.7 ± [10.1–31.6]	0.004 **	30,410.7 ± [11,781.2–52,059.7] vs. 28,833.2 ± [15,409.3–48,093.1]	0.009 **
Pre-obese vs. underweight	22.6 ± [9.3–33.1] vs. 20.7 ± [10.1–31.6]	<0.001 ***	32,904.4 ± [13,640.6–51,346.1] vs. 28,833.2 ± [15,409.3–48,093.1]	<0.001 ***
Obese vs. underweight	23.3 ± [11.0–35.3] vs. 20.7 ± [10.1–31.6]	<0.001 ***	32,947.4 ± [13,618.6–52,359.9] vs. 28,833.2 ± [15,409.3–48,093.1]	<0.001 ***
Pre-obese vs. normal weight	22.6 ± [9.3–33.1] vs. 22.1 ± [8.9–35.1]	0.01 *	32,904.4 ± [13,640.6–51,346.1] vs. 30,410.7 ± [11,781.2–52,059.7]	0.006 **
Obese vs. normal weight	23.3 ± [11.0–35.3] vs. 22.1 ± [8.9–35.1]	0.3	32,947.4 ± [13,618.6–52,359.9] vs. 30,410.7 ± [11,781.2–52,059.7]	0.01 *

The Kruskal–Wallis rank sum test, with a post hoc analysis, was performed with Dunn’s test. Statistical significance was attributed as * *p* < 0.05; ** *p* < 0.01; or *** *p* < 0.001. Abbreviations: body mass index (BMI); CO_2_ production by food (CO_2_); H_2_O consumption by food production (H_2_O).

**Table 3:** Generalized linear model for BMI and CO_2,_ and BMI and H_2_O.

**Coefficients**	**Estimate**	**Std. Error**	**z-Value**	***p*-Value**
Intercept	−1.19308	0.34785	−3.430	<0.001 ***
BMI (kg/m^2^)	0.04782	0.01239	3.859	<0.001 ***
CO_2_ (eq/week)	0.08192	0.00996	8.226	<0.001 ***
Null deviance: 3021.5 on 3532 degrees of freedom Residual deviance: 2929.2 on 3530 degrees of freedom AIC: 2935.2				
**Coefficients**	**Estimate**	**Std. Error**	**z-Value**	***p*-Value**
Intercept	−2.20135	0.34748	−6.335	<0.001 ***
BMI (kg/m^2^)	0.03951	0.01254	3.151	0.001 **
H_2_O (L/week)	0.00009	0.00007	13.922	<0.001 ***
Null deviance: 3021.5 on 3532 degrees of freedom Residual deviance: 2929.2 on 3530 degrees of freedom AIC: 2780.7				

Statistical significance was attributed as ** *p* < 0.01; or *** *p* < 0.001. Abbreviations: body mass index (BMI); CO_2_ production by food (CO_2_); H_2_O consumption by food production (H_2_O).

**Table 4:** Comparisons between the occupation groups for CO_2_ and H_2_O.

	CO_2_ (eq/Week)		H_2_O (L/Week)	
Occupation Groups				
	Median ± [minimum–maximum value]	*p*-value	Median ± [minimum–maximum value]	*p*-value
Overall population		<0.001		<0.001
Unemployed vs. retired	22.2 ± [10.3–32.8] vs. 24.3 ± [9.7–32.9]	0.02 *	32,180.9 ± [17,030.0 –49,159.0] vs. 36,296.1 ± [14,859.0–47,447.0]	0.01 *
Unemployed vs. students	22.2 ± [10.3–32.8] vs. 20.9 ± [9.2–33.1]	0.001 **	32,180.9 ± [17,030.0–49,159.0] vs. 28,816.0 ± [13,524.0–48,172.0]	<0.001
Unemployed vs. employed	22.2 ± [10.3–32.8] vs. 22.5 ± [9.0–35.4]	0.9	32,180.9 ± [17,030.0–49,159.0] vs. 31,382.3 ± [11,781.0–52,360.0]	0.9
Retired vs. students	24.3 ± [9.7–32.9] vs. 20.9 ± [9.2–33.1]	<0.001 ***	36,296.1 ± [14,859.0–47,447.0] vs. 28,816.0 ± [13,524.0–48,172.0]	<0.001 ***
Retired vs. employed	24.3 ± [9.7–32.9] vs. 22.5 ± [9.0–35.4]	0.006 **	36,296.1 ± [14,859.0–47,447.0] vs. 31,382.3 ± [11,781.0–52,360.0]	0.001 **
Students vs. employed	20.9 ± [9.2–33.1] vs. 22.5 ± [9.0–35.4]	<0.001 ***	28,816.0 [13,524.0–48,172.0] vs. 31,382.3 ± [11,781.0–52,360.0]	<0.001 ***

Values are expressed as median, minimum, and maximum values (median ± [min.–max. value]) for continuous variables. The Kruskal–Wallis rank sum test, with a post hoc analysis, was performed with Dunn’s test. Statistical significance was attributed as * *p* < 0.05; ** *p* < 0.01; or *** *p* < 0.001. Abbreviations: CO_2_ production by food (CO_2_); H_2_O consumption by food production (H_2_O).

**Table 5:** Comparison between the occupation groups for CO_2_ and H_2_O.

	CO_2_ (eq/Week)		H_2_O (L/Week)	
MEDAS Groups				
	Median ± [minimum–maximum value]	*p*-value	Median ± [minimum–maximum value]	*p*-value
Overall population		<0.001 ***		<0.001 ***
Low adherence vs. high adherence	23.6 ± [9.2–35.3] vs. 21.0 ± [10.0–32.0]	<0.001 ***	36,364.5 ± [3618.6–52,359.9] vs. 27,296.7 ± [13,640.6–46,965.8]	<0.001 ***
Medium adherence vs. high adherence	22.4 ± [8.9–33.9] vs. 21.0 ± [10.0–32.0]	<0.001 ***	31,015.5 ± [11,781.2–51,346.1] vs. 27,296.7 ± [13,640.6–46,965.8]	<0.001 ***
Medium adherence vs. low adherence	22.4 ± [8.9–33.9] vs. 23.6 ± [9.2–35.3]	<0.001 ***	31,015.5 ± [11,781.2–51,346.1] vs. 36,364.5 ± [3618.6–52,359.9]	<0.001 ***

Values are expressed as median, minimum, and maximum values (median ± [min.–max. value]) for continuous variables. The Kruskal–Wallis rank sum test, with a post hoc analysis, was performed with Dunn’s test. Statistical significance was attributed as *** *p* < 0.001. Abbreviations: CO_2_ production by food (CO_2_); H_2_O consumption by food production (H_2_O); adherence to the Mediterranean diet (MEDAS).

**Table 6:** Comparisons between occupation groups for the MEDAS score.

	MEDAS Score	
	Median ± [minimum–maximum value]	*p*-value
Overall population		0.02 *
Unemployed vs. retired	7.0 ± [1.0–8.0] vs. 7.0 ± [1.0–8.0]	0.9
Unemployed vs. students	7.0 ± [1.0–8.0] vs. 7.0 [1.0–9.0]	0.02 *
Unemployed vs. employed	7.0 ± [1.0–8.0] vs. 7.0 [1.0–9.0]	0.3
Retired vs. students	7.0 [1.0–9.0] vs. 7.0 [1.0–9.0]	0.8
Retired vs. employed	7.0 ± [1.0–8.0] vs. 7.0 [1.0–9.0]	0.9
Students vs. employed	7.0 [1.0–9.0] vs. 7.0 [1.0–9.0]	0.3

Values are expressed as median, minimum, and maximum values (median ± [min.–max. value]) for continuous variables. The Kruskal–Wallis rank sum test, with a post hoc analysis, was performed with Dunn’s test. Statistical significance was attributed as * *p* < 0.05. Abbreviations: adherence to the Mediterranean diet (MEDAS).

**Table 7:** Multiple linear regression based on the stepwise method. Statistical significance was attributed as * *p* < 0.05; ** *p* < 0.01; or *** *p* < 0.001. Abbreviations: body mass index (BMI); CO_2_ production by food (CO_2_); H_2_O consumption by food production (H_2_O); adherence to the Mediterranean diet (MEDAS).

	BMI (kg/m^2^)	CO_2_ (eq/Week)	H_2_O (L/Week)	Age	Weekly Food Cost (EUR/Week)
MEDAS score	*r*^2^ = 0.70	*r*^2^ = 0.97	*r*^2^ = 0.87	*r*^2^ = 0.09	*r*^2^ = 0.98
	*p* = 0.004 **	*p* = 0.01 *	*p* < 0.001 ***	*p* = 0.7	*p* = 0.006 **

**Table 8:** Comparisons between MEDAS groups for the weekly food costs.

	Weekly Food Cost	
	Median ± [minimum–maximum value]	*p*-value
Overall population		<0.001 ***
Low adherence vs. medium adherence	97.2 ± [46.2–137.9] vs. 94.2 ± [44.8–136.9]	0.004 **
Low adherence vs. high adherence	97.2 ± [46.2–137.9] vs. 90.0 ± [49.7–128.6]	<0.001 ***
Medium adherence vs. high adherence	94.2 ± [44.8–136.9] vs. 90.0 ± [49.7–128.6]	<0.001 ***

Values are expressed as median, minimum, and maximum values (median ± [min.–max. value]) for continuous variables. Weekly food cost values are expressed as EUR/week. The Kruskal–Wallis rank sum test, with a post hoc analysis, was performed with Dunn’s test. Statistical significance was attributed as ** *p* < 0.01 or *** *p* < 0.001. Abbreviations: adherence to the Mediterranean diet (MEDAS).

**Table 9:** Comparisons between organic and non-organic markets.

	Organic Market *n* = 137 (F = 114)	Non-Organic Market *n* = 3396 (F = 2576)	
Parameters			
	Median ± [minimum–maximum value]	Median ± [minimum–maximum value]	*p*-value
BMI (kg/m^2^)	22.3 ± [16.8–45.3]	23.3 ± [14.0–51.5]	<0.001 ***
Weight (kg)	61.0 ± [42.0–116.0]	65.0 ± [34.0–154.0]	0.02 *
MEDAS score	8.0 ± [3.0–13.0]	7.0 ± [1.0–14.0]	<0.001 ***
Weekly food cost (EUR/week)	88.0 ± [57.1–141.6]	80.5 ± [44.5–138.1]	<0.001 ***
CO_2_ (eq/week)	15.9 ± [8.1–25.0]	18.6 ± [8.6–35.4]	<0.001 ***
H_2_O (L/week)	27,487.0 ± [13,753.4–46,015.2]	28,689.2 ± [11,781.2–52,359.9]	0.3

Values are expressed as median, minimum, and maximum values (median ± [min.–max. value]) for continuous variables. A Mann–Whitney U test was performed. Statistical significance was attributed as * *p* < 0.05 or *** *p* < 0.001. Abbreviations: body mass index (BMI); CO_2_ production by food (CO_2_); H_2_O consumption by food production (H_2_O); adherence to the Mediterranean diet (MEDAS).

## Data Availability

The data presented in this study are available on request from the corresponding author.
